# Synthesis and characterization of bevacizumab-functionalized nanoliposomes loaded with regorafenib and fluorescent carbon dots for triple-negative breast cancer theranostics

**DOI:** 10.1039/d6ra03364d

**Published:** 2026-05-26

**Authors:** Armita Aryanmanesh, Abolghasem Abbasi Kajani

**Affiliations:** a Department of Biotechnology, Faculty of Biological Science and Technology, University of Isfahan Isfahan 81746-73441 Iran agh.abbasi@bio.ui.ac.ir agh.abasi@gmail.com +98-3137932342 +98-3137934401

## Abstract

A novel theranostic nanoplatform was developed by a facile and green approach for targeted drug delivery and high-quality fluorescence imaging of triple-negative breast cancer cells (TNBCs). Innovatively synthesized fluorescent carbon dots (CDs) obtained by a hydrothermal method with a zeta potential of −18.4 mV had uniform sizes of <20 nm and high fluorescence quantum yield of 27.3% at 460 nm. Nanoliposomes (NLPs) were synthesized by a thin-film hydration method and loaded with CDs and regorafenib (CR-NLPs) and covalently functionalized with the bevacizumab antibody (BCR-NLPs) by the EDC–NHS method. Monodispersed and high colloidal BCR-NLPs showed a semi-spherical shape in the size range of 100–200 nm and zeta potential of −44.3 mV. HPLC of regorafenib revealed a maximum loading efficiency of 82% and sustained release of 96.4% during 120 h with the first-order kinetic model. The *in vitro* MTT assay showed the high biocompatibility of CDs, NLPs, CR-NLPs, and BCR-NLPs after 48 h exposure to human fibroblast cells at 200 µg mL^−1^, with cell viability of 91.4 ± 1.7, 92.2 ± 2.4, 91.5 ± 3, and 88.7 ± 3.1%, respectively. Under the same conditions, potent and specific anticancer activity was observed on MDA-MB-231 cells for CR-NLPs and BCR-NLPs with cell viability of 64.5 ± 1.7 and 47.6 ± 2.3%, respectively. The high-quality fluorescence imaging of MDA-MB-231 cancer cells was also obtained *in vitro* using BCR-NLPs. The overall results confirmed the high potential of BCR-NLPs for cancer theranostic application.

## Abstract 2

A novel theranostic nanoplatform based on bevacizumab functionalized nanoliposomes incorporated with carbon dots for the targeted and controlled delivery of regorafenib and fluorescence imaging of triple negative breast cancer cells.

## Graphical abstract

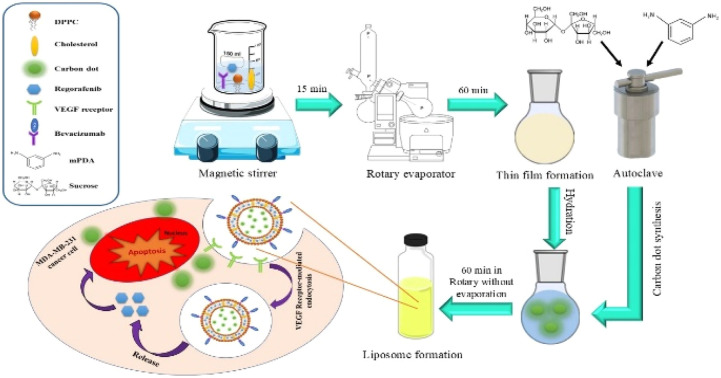

## 1. Introduction

Breast cancer (BC) is one of the most common cancers in women. BC remains one of the leading causes of cancer-related deaths.^1^ The control of BC is difficult due to the increased prevalence, resistance to drug effects, potential metastases and genomic disruption. Among different types of BC, triple-negative BC (TNBC) is characterized by the lack of estrogen, progesterone, and human epidermal growth factor receptor 2 (HER2).^2^ Despite important advances in the surgical, radiotherapy, and chemotherapy modalities, there is no efficacious targeted therapy for this cancer subtype, and major challenges, such as poor drug selectivity, severe systemic side effects, and drug resistance, have not been solved.^3–5^

Regorafenib is a tyrosine kinase inhibitor (TKI). It plays an important part in the inhibition of tumor growth and progression by the blockade of a wide-range of receptors involved in angiogenesis, cell proliferation, and survival.^6^ However, the poor pharmacological properties of this TKI, especially its low solubility and permeability, have limited its direct application due to heterogeneous distribution in tissues, *in vivo* metabolism and degradation, and systemic toxicity. Therefore, improvement of the pharmacokinetics and bioavailability of regorafenib is essential to achieve an effective therapeutic response.^7,8^

Nanobiotechnology, with the development of engineered nanoparticles (NPs) of 1–100 nm in size, has emerged as a promising approach to overcome the limitations of conventional therapies.^9^ Nanocarriers-based drug delivery can lead to efficient crossing through complex biological barriers and overcome the low stability, inefficient and nonspecific biodistribution, rapid metabolism, and systemic toxicity of drugs.^10^ Moreover, the high surface-to-volume ratio of nanocarriers and their appropriate surface functionalization provides targeted and controlled delivery of therapeutics.^11^

The therapeutic efficacy of nanocarriers can also be increased further by active targeting using small molecules, antibodies, peptides, and aptamers, or by passive targeting using the characteristic phenomenon of enhanced permeability and retention (EPR) in tumors.^12,13^ Among different active targeting ligands, antibodies are considered to be the most common targeting agents due to high selectivity, specificity and binding affinity.^13^ Bevacizumab is a humanized monoclonal antibody with the trade name of Avastin®. It is commonly used in combination with chemotherapeutic agents for cancers such as colorectal, cervical, renal, glioblastoma, and advanced hepatocellular carcinoma.^14–16^ Bevacizumab was the first anti-angiogenic agent approved by the USA Food and Drug Administration (FDA). It inhibits the VEGF expression of cancer cells and finally leads to cell necrosis.^16,17^ The combined administration of a TKI with bevacizumab has been suggested as a solution for overcoming the common problem of resistance to anti-VEGF drugs and also for improving the efficacy of cancer therapy through their synergistic effects.^18^

Nanoliposomes (NLPs) are one of the most widely used nanocarriers in cancer research and clinical applications due to their high biocompatibility, biodegradability, and sustained release of drugs.^7,19^ NLPs, which are composed of one or more phospholipid layers, show great similarity to cell membranes.^20^ This structural feature allows for simultaneous loading of hydrophilic and lipophilic drugs in their inner aqueous compartment and lipid bilayers, respectively, and their efficient cellular uptake.^21^

**Scheme 1:**
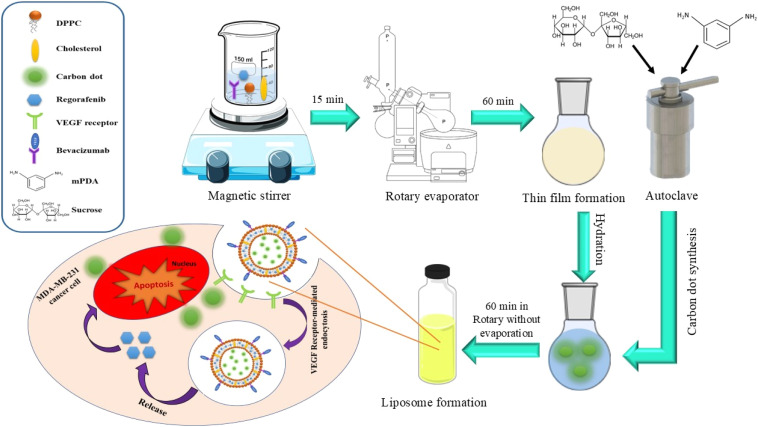
Stepwise synthesis and *in vitro* study of BCR-NLPs (schematic).

In the present study, fluorescent carbon dots (CDs) were first synthesized by a green and simple hydrothermal method. NLPs were then synthesized by a thin-film hydration (TFH) method. NLPs were loaded with CDs and regorafenib (CR-NLPs) and functionalized by bevacizumab antibody (BCR-NLPs). After physicochemical characterization by appropriate methods, the biocompatibility and anticancer activity of NLPs were investigated, *in vitro*, on human fibroblast (HFB) and MDA-MB-231 cell lines, respectively. Finally, the potential use of BCR-NLPs for the fluorescence imaging of MDA-MB-231 cancer cells was evaluated *in vitro* (Scheme 1). This is the first report on the development of a theranostic nanoliposome incorporating regorafenib, bevacizumab, and CDs for the targeted therapy and fluorescent imaging of TNBC.

## 2. Materials and methods

### 2.1. Materials

1,2-Dipalmitoylphosphatidylcholine (DPPC), cholesterol, chloroform (99.9%), palmitic acid (PA), 1-ethyl-3-(3-dimethylaminopropyl)carbodiimide (EDC), *N*-hydroxysuccinimide (NHS), glycine, 12–14 kDa dialysis bag, and 3-(4,5-dimethylthiazol-2-yl)-2,5-diphenyltetrazolium bromide (MTT) were purchased from Sigma-Aldrich (Germany). Sucrose, methanol (99.9%), dimethyl sulfoxide (DMSO), fluorescein, and *m*-phenylenediamine (*m*PDA), were obtained from Merck (Darmstadt, Germany). DMEM–F12 (high glucose), trypsin–0.03% (w/v) EDTA solution, antibiotic (penicillin–streptomycin) solution, and fetal bovine serum (FBS) were from Gibco (Grand Island, NY, USA). HFB and MDA-MB-231 cell lines were obtained from Isfahan University of Medical Science (Isfahan, Iran). Bevacizumab was a gift from AryoGen Pharmed (Tehran, Iran). Regorafenib was a gift from Nanoalvand Pharmaceuticals (Tehran, Iran).

### 2.2. Synthesis of CDs and BCR-NLPs

Fluorescent CDs were synthesized by a hydrothermal method using sucrose as the carbon source and *m*PDA as the nitrogen source. Briefly, an aqueous solution of 0.1 M sucrose and 0.05 M *m*PDA (final volume of 20 mL) was prepared and transferred into a Teflon-lined autoclave, sealed, and heated at 150 °C for 3 h. After cooling to room temperature, the resultant colloidal solution was centrifuged at 10 000 rpm for 15 min to precipitate and remove impurities. Then, colloidal CDs were separated from unreacted precursors by antisolvent precipitation using isopropanol and centrifugation at 10 000 rpm for 30 min. Finally, the purified CDs were filtered and freeze-dried for subsequent studies.

NLPs were then synthesized by the TFH method according to a previous report with some modifications.^22^ Briefly, an organic phase containing 10 mg of cholesterol and 40 mg of DPPC in 5 mL of chloroform was prepared and added to a 50 mL round-bottom flask. This was followed by solvent evaporation using a rotary evaporator under a vacuum at 45 °C for 2 h to form a thin lipid layer. Then, 5 mL of PBS (pH 7.4) was added to the flask under vigorous shaking to hydrate the thin layer, followed by incubation at 45 °C for 1 h to prepare and stabilize the NLPs. For the synthesis of CR-NLPs, regorafenib was added to the initial organic phase, while CDs were incorporated into NLPs during the hydration step through PBS (pH 7.4).

Bevacizumab functionalization was performed based on previous reports.^23,24^ Briefly, 5 mL of PA solution (1 mM in chloroform) was prepared and added to a 50 mL round-bottom flask, followed by solvent evaporation using a rotary evaporator under a vacuum at 45 °C for 2 h. The resultant lipid film was then hydrated by the addition of 50 mL of PBS (pH 7.4) under shaking at 60 °C for 10 min. Subsequently, EDC (100 µL, 0.1 M) and NHS (500 µL, 0.1 M) were added to the flask and incubated for 30 min at 25 °C, before addition of the antibody and incubation under the same conditions for 30 min. Finally, glycine solution (1000 µL, 100 mM) was added to the solution and incubated again for 4 h to block unreacted functional groups. The resultant PA–antibody conjugates were used at a weight ratio of 1 : 50 cholesterol : DPPC for the synthesis of BCR-NLPs.

### 2.3. Characterization of NLPs

The size and morphology of CDs and NLPs were characterized using high-resolution transmission electron microscopy (HR-TEM; Philips EM 208s). The hydrodynamic size distribution and surface charge of CDs, NLPs, R-NLPs, and BCR-NLPs were measured using a SZ-100 NP analyzer (Horiba, Kyoto, Japan). The surface chemistry of NPs was studied using Fourier transform infrared (FTIR) spectroscopy (FTIR 6300, Jasco, Japan) in a wavenumber range of 4000–400 cm^−1^. The optical properties of CDs were characterized using UV-vis spectroscopy (Carry 100, Agilent Technologies) and fluorescence spectroscopy (RF-5000, Shimadzu). The fluorescence quantum yield (QY) of CDs was calculated by the following equation using fluorescein (QY = 95%) as a standard:QY_*x*_ = QY_std_(*I*_*x*_/*I*_std_)(*R*_std_/*R*_*x*_)(*n*_*x*_^2^/*n*_std_^2^)

### 2.4. Loading and release studies

An HPLC system (Knauer, Azura, Germany) equipped with a Kromasil C18 column (250 mm × 4.6 mm) was used to study the loading efficiency (LE) and release profile of regorafenib based on previous reports.^25,26^ Briefly, different concentrations (0.5, 1, 2.5, and 5 mg) of regorafenib were used in the synthesis of NLPs. The resultant R-NLPs were then centrifuged at 10 000*g* for 10 min to precipitate R-NLPs. The concentration of regorafenib in the supernatant was measured through a mobile phase of water : acetonitrile (50 : 50, v/v) at a flow rate of 1 mL min^−1^ and an absorbance wavelength of 261 nm at 25 °C. The LE of NLPs was calculated using the following equation:^27,28^
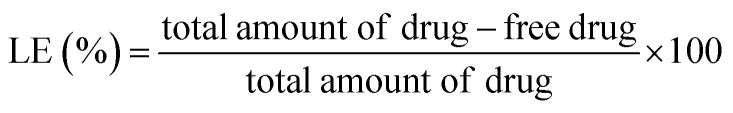


The release kinetics of regorafenib from R-NLPs were studied using dialysis bags (MWCO = 12–14 kDa) soaked in PBS and HPLC measurement of the drug concentration at different intervals (0, 12, 24, 48, 72, 96, and 120 h).

### 2.5. *In vitro* assays

The biocompatibility of NPs was first studied on HFB cells by the MTT assay according to a previous report.^14^ For this purpose, 10^4^ cells were first seeded on each well of a 96-well plate and incubated at 37 °C and 5% CO_2_ in a humidified incubator for 24 h. Then, cells were treated with different concentrations (50, 100, and 200 µg mL^−1^) of NLPs for 48 h. The medium was then discarded and replaced with 100 µL of MTT solution (0.5 mg mL^−1^ in medium). After incubation for 4 h under the same conditions, the medium was removed again and the precipitated formazan crystals were dissolved in 100 µL of DMSO before absorbance measurement at 570 nm. Then, cell viability was calculated as the ratio of the absorbance value of each treatment compared with the control value. The potential anticancer activity of NLPs, R-NLPs, BR-NLPs and BCR-NLPs was also studied on MDA-MB-231 cells using the MTT assay with the same procedure. Changes in the morphology of HFB cells were also monitored by an optical microscope after exposure with NLPs. The potential of BCR-NLPs for the fluorescence imaging of MDA-MB-231 cells was also studied *in vitro* using an inverted microscope (Nikon, Eclipse Ti-U) after incubation with 200 µg mL^−1^ of BCR-NLPs for 6 h.

### 2.6. Statistical analyses

All *in vitro* studies were conducted in triplicate. Results are the mean ± standard deviation (SD). Student's *t*-test (Microsoft Excel, Microsoft, USA) was used for data analyses. *P* < 0.05 were considered significant.

## 3. Results and discussion

### 3.1. Synthesis and characterization of CDs and BCR-NLPs

CDs have recently attracted much attention for various biomedical applications, especially bioimaging, due to their excellent biocompatibility, high fluorescence efficiency, as well as the simple and relatively low-cost synthesis.^29^ The hydrothermal synthesis of CDs using various organic sources, including carbohydrates, proteins, and other biomolecules, has been reported recently.^30^ Different sugars, such as glucose, fructose, xylose, and sucrose, have been used for the synthesis of CDs by various methods, including hydrothermal,^31,32^ solvothermal,^33^ microwave,^34^ plasma treatment,^35^ and pyrolysis.^36^ Sucrose was used in the present study as a food-grade, abundant and cheap source of CDs synthesis *via* a green hydrothermal method. Considering the relevant results of our previous reports^29,37^ regarding the use of *m*PDA for the N-doping of CDs, this aromatic diamine was used in the hydrothermal reaction as the nitrogen source.

**Fig. 1:**
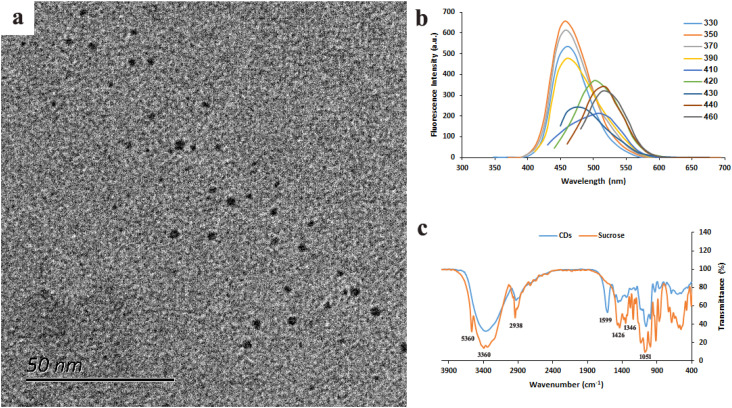
TEM (a), fluorescence spectra (b), and FTIR spectra (c) of CDs.

Based on initial experiments, the optimum condition was determined to be the hydrothermal treatment of a reaction mixture of 0.1 M sucrose and 0.05 M *m*PDA at 150 °C for 3 h. TEM showed that this simple and one-step method led to the synthesis of monodispersed and semispherical CDs with uniform sizes of <10 nm (Fig. 1a). The resultant CDs had high colloidal stability and water dispersibility with a zeta potential of −18.4 (Fig. S1). CDs displayed excitation-dependent fluorescence emission and a high quantum yield (QY) of 27.3% at 460 nm with maximum excitation at 350 nm (Fig. 1b). XRD analysis (Fig. S2) showed a broad diffraction peak centered at a 2*θ* angle of ∼12.5°, indicating an amorphic structure of CDs. FTIR spectroscopy (Fig. 1c) revealed absorption peaks at 3560 cm^−1^, 3360 cm^−1^, 2938 cm^−1^, 1599 cm^−1^, 1426 cm^−1^, 1346 cm^−1^, and 1051 cm^−1^, which corresponded to O–H vibration, N–H stretching, C–H stretching, C=C stretching, C–O–C stretching, C–N stretching, and C–O stretching, respectively.^30,37^ The presence of different functional groups on the surface of CDs was a special advantage for their hydrophilicity and subsequent surface functionalization.

In the next step, BCR-NLPs were synthesized and characterized for cancer theranostic application. In addition to conventional methods, such as TFH, solvent injection, and reverse-phase evaporation, some new methods have been reported for the synthesis of NLPs, such as the microfluidic-assisted synthesis and condensed gas technology.^38^ Among these, TFH is considered to be one of the most widely used methods due to its simplicity, cost-effectiveness, and scalability.^39^ The antibody targeting of NLPs was carried out by covalent conjunction of bevacizumab antibody to EDC/NHS-activated PA and its subsequent incorporation into a lipid membrane during NLPs synthesis. Carbodiimide coupling led to direct formation of amide bonds between carboxylic acid groups on the surface of NLPs and the free amine groups of the antibodies without the need for the commonly used lengthy and toxic linkers that increase the hydrodynamic size of NLPs and limit the biomedical applications of NLPs.^40^

**Fig. 2:**
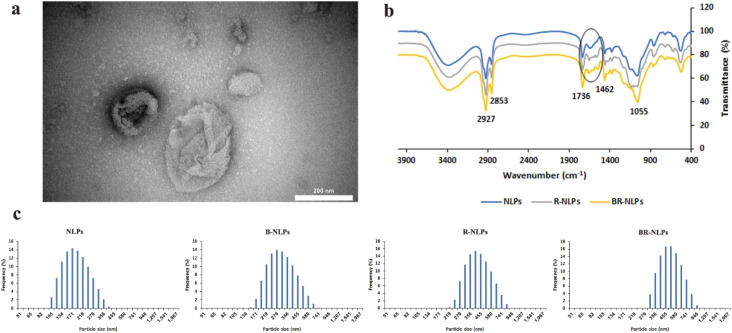
TEM of BCR-NLPs (a). FTIR spectra (b) and hydrodynamic sizes (c) of different NLPs.

The synthesis of BCR-NLPs was evaluated using TEM, FTIR spectroscopy, DLS and the zeta potential. TEM showed the synthesis of monodispersed and spherical BCR-NLPs in the appropriate size range of 100–200 nm (Fig. 2a). A stepwise study of the surface chemistry of NLPs using FTIR spectroscopy confirmed their functionalization (Fig. 2b). The appearance of new peaks around 1500–1650 cm^−1^ could be attributed to the amide I and amide II bands originating from the covalent attachment of bevacizumab antibody to NLPs.^41^ Accordingly, a significant change in the surface charge of NLPs was also observed after antibody conjugation and drug loading (Fig. S3), such that zeta potential values of −59.8, −46.2, and −44.3 were obtained for NLPs, B-NLPs, and BCR-NLPs, respectively. DLS also showed increased hydrodynamic sizes of NLPs following drug loading and antibody conjugation (Fig. 2c). An average hydrodynamic size of 183.50, 307.1, 419.1, and 495 nm and PDI of 0.63, 0.9, 0.85, and 0.93 obtained for NLPs, B-NLPs, CR-NLPs, and BCR-NLPs, respectively, indicated a suitable size of functionalized NLPs, in accordance with TEM results.

### 3.2. Loading and release studies

**Fig. 3:**
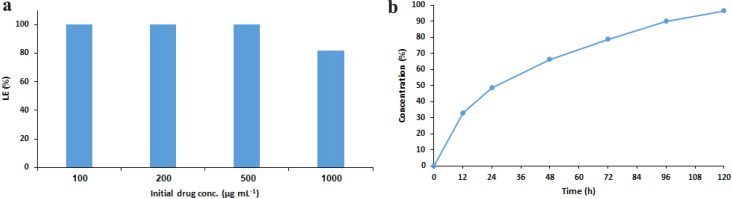
The loading efficiency (%) of regorafenib in NLPs (a) and its time-dependent release (%) in PBS (b).

The efficiency of regorafenib loading in NLPs was calculated based on the concentration of the drug in the supernatant. HPLC studies showed that, in the presence of initial concentrations of 0.1, 0.2, and 0.5 mg mL^−1^ of regorafenib, 100% of the drug was loaded in NLPs. However, an LE of 82% obtained using an initial concentration of 1 mg mL^−1^ of regorafenib (Fig. 3a) led to a maximum encapsulation of 0.82 mg mL^−1^ of the drug. The use of higher concentrations of regorafenib led to the significant instability and agglomeration of NLPs, as well as the accumulation of free drugs and formation of an organic layer in colloids. The high lipophilic nature of regorafenib is the main cause of its inefficiency and an important obstacle for its biomedical application. Hence, our results confirmed the high potential of NLPs as appropriate carriers for this anticancer drug, as well as for other similar therapeutic agents.

HPLC of *in vitro* drug release by a dialysis method showed a first-order kinetic model for regorafenib release from NLPs into PBS (pH 7.4). Results (Fig. 3b) showed an initial release of 33% during 12 h, followed by controlled release at a lower rate such that a cumulative release of 96.4% occurred over 120 h. These results indicated the ability of NLPs for the sustained release of a drug, which further confirmed the suitability of NLPs as appropriate drug delivery carriers of regorafenib.

### 3.3. *In vitro* cell studies

**Fig. 4:**
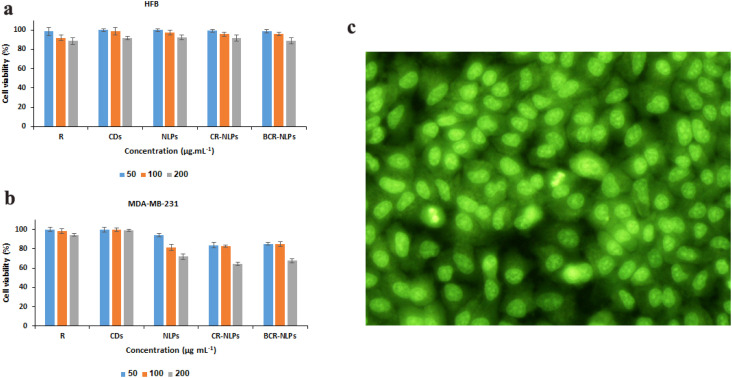
Viability percentages of HFB (a) and MDA-MB-231 (b) cells after treatment with different concentrations of regorafenib (R), CDs, NLPs, CR-NLPs, and BCR-NLPs for 48 h, and fluorescence imaging of MDA-MB-231 cells after treatment with BCR-NLPs for 6 h (c).

The potential cytotoxicity of different nanoformulations was first studied on HFB cells by the MTT assay. The high biocompatibility of CDs, NLPs, CR-NLPs, and BCR-NLPs was observed with viability percentages of 91.4 ± 1.7, 92.2 ± 2.4, 91.5 ± 3, and 88.7 ± 3.1%, respectively, obtained after 48 h exposure with 200 µg mL^−1^ of these NPs. Under the same conditions, a cell viability of 88.51 ± 3.47% was obtained for regorafenib (Fig. 4a). On the other hand, the MTT assay on MDA-MB-231 cells revealed the significant anticancer activity of CR-NLPs, and BCR-NLPs with viability percentages of 64.5 ± 1.7 and 47.6 ± 2.3%, respectively, under the same conditions. Considerable anticancer activity was not observed in the same conditions for NLPs, CDs, or regorafenib, with viability percentages of 89.8 ± 2.7, 98.8 ± 0.9 and 94.4 ± 1.4%, respectively (Fig. 4b). These results suggested the preliminary selectivity and significant anticancer activity of BCR-NLPs, but also confirmed the effect of functionalization with regorafenib antibody on the improved anticancer effect of BCR-NLPs. The enhanced cytotoxicity of BCR-NLPs compared with that of CR-NLPs could be attributed to two mechanisms. First, bevacizumab binds to VEGF receptors overexpressed on MDA-MB-231 cells, facilitating the internalization of NPs through receptor-mediated endocytosis. Second, regorafenib and bevacizumab target different pathways involved in tumor growth and angiogenesis, leading to a combined effect greater than the sum of their individual contributions. Together, these mechanisms explain the superior anticancer activity of the targeted formulation.

In accordance with the results of the MTT assay, microscopic observation also showed a significant change in the proliferation and morphology of MDA-MB-231 cells following different treatments (Fig. S4). Microscopic evaluation of HFB cells after exposure with different NPs also confirmed the results of the MTT assay (Fig. S5). Interestingly, exposure to BCR-NLPs for 6 h led to intensive fluorescence emission and high-quality imaging of MDA-MB-231 cells (Fig. 4c), indicating high potential of BCR-NLPs for theranostic application.

## 4. Discussion

Use of nanocarriers is an advanced and efficient approach to improve the stability and bioavailability of hydrophobic drugs. The specificity and selectivity of drug delivery nanocarriers can be further improved by their targeting using appropriate ligands, especially cell-surface-recognition antibodies. Moreover, drug release can also be monitored by the incorporation of an appropriate agent, such as the fluorescent CDs. Thus, these approaches were used to develop a novel liposome-based nanocarrier for the targeted delivery of regorafenib to the TNBC cells of MDA-MB-231. The targeting of BC cells by bevacizumab monoclonal antibody provides the selective delivery of regorafenib but also can reduce angiogenesis and induce apoptosis in tumors *via* specific binding to VEGF receptors.^42,43^ Therefore, synergistic effects can be expected by the binding of bevacizumab antibody to R-NLPs.

The renal and/or hepatic clearance of drugs, antibodies, and tracers is another challenge towards advanced and efficient bioimaging and therapy of diseases.^44,45^ With regard to the fast renal clearance of CDs due to their ultra-small sizes,^46^ their encapsulation in appropriate carriers is necessary to obtain the appropriate efficacy.^47^ The conjugation of bevacizumab to NLPS has been reported to enhance the physical stability of antibody in serum, leading to synergistic anti-angiogenic effects in combination with ketoconazole.^48^ Similar results have also been reported by the combined administration of bevacizumab and paclitaxel in patients with metastatic BC.^49^ Dual targeting of TNBC tumors using antibodies-conjugated liposomal doxorubicin against intercellular adhesion molecule-1 (ICAM1) and the epithelial growth factor receptor (EGFR) has also been reported to improve the tumor targeting and antitumor activity of liposomes.^5^ The combined use of two anti-VEGFRs agents of lenvatinib and regorafenib has also been reported to represent a synergistic anti-angiogenic effect on MCF7 and MDA-MB-231 cell lines.^50^ Therefore, the rational design of BCR-NLPs can: (a) improve the *in vivo* stability of bevacizumab and CDs as well as biodistribution of regorafenib; (b) lead to the synergistic and selective therapeutic effect of bevacizumab and regorafenib; (c) enable simultaneous monitoring of drug delivery by fluorescence imaging.

Although the high biocompatibility of BCR-NLPs on fibroblasts is encouraging, this does not fully exclude off-target effects on other normal cells and tissues. Future studies will include toxicity assessment on hepatocyte and renal cell lines, as well as evaluation of hemocompatibility and immunogenicity, to better understand the safety profile of this nanoplatform.

## 5. Conclusions

The efficient therapeutic potential of a tyrosine kinase inhibitor (regorafenib) in combination with an anti-VEGF antibody (bevacizumab) in a single targeted liposomal carrier was demonstrated in the present study for the first time. The incorporation of CDs into liposomal carriers was used for demonstration of drug delivery and the high-quality fluorescence imaging of TNBC cells.

Fluorescent CDs with high QY of 27.3% were synthesized innovatively from sucrose by a green and one-pot method at low temperature (150 °C) within 3 h, which is simpler and more cost-effective than many previously reported methods. Compared with conventional imaging agents such as organic dyes and semiconductor quantum dots, CDs offer several key advantages for theranostic applications. CDs exhibit excellent biocompatibility and low toxicity due to their carbon-based composition, unlike the heavy metal-containing quantum dots. CDs show superior photostability and resistance to photobleaching compared with organic dyes, enabling long-term fluorescence imaging without significant signal decay. The facile and green synthesis of high colloidal CDs from low-cost precursors makes them reproducible and potentially translatable to clinical applications. Use of a simple, reliable, and controllable hydrothermal method for the one-step synthesis of CDs further facilitates the preparation of CDs in an efficient, ecofriendly, and large-scale manner. In summary, CDs offer a unique combination of low toxicity, high photostability, green synthesis, and excellent fluorescence properties. These advantages make them clearly superior to traditional organic dyes and quantum dots for long-term and *in vivo* theranostic applications, fully justifying their selection in our platform.

Use of the TFH method as a simple and cost-effective method for the synthesis of NLPs, provided a high (82%) loading efficiency of hydrophobic regorafenib into the lipid bilayer with the subsequent release with appropriate kinetics. The method also allowed us to incorporate the bevacizumab antibody onto the liposome surface through EDC/NHS chemistry. This well-established method has been employed in the numerous studies to prepare targeted liposomes for cancer therapy and, thus, for initial proof-of-concept investigations in academic settings, TFH remains a rational and effective choice. Therefore, the TFH method was selected because it offers simplicity, flexibility, and high encapsulation efficiency, which are required for initial theranostic development.

Bevacizumab was conjugated to palmitic acid-incorporated nanoliposomes using the EDC/NHS method. The EDC/NHS reaction forms stable amide bonds between the carboxyl groups on the NP surface and primary amines on the antibody. These covalent linkages are resistant to hydrolysis and maintain the antibody–NP association under physiological conditions. While we did not perform direct quantification of conjugation efficiency. The combined evidence from zeta potential changes, FTIR spectroscopy, and the enhanced biological activity of BCR-NLPs compared with non-targeted controls collectively confirmed that bevacizumab had been conjugated with preserved functionality. The EDC/NHS method, despite its limitations in conjugation efficiency and orientation control, provided sufficient antibody loading and functional activity for *in vitro* proof-of-concept studies.

Our multifunctional nanoplatform improves on previous targeted drug delivery systems for TNBC that mainly focus on a single drug or a drug and a targeting agent. Interestingly, our developed nanoplatform integrated all these agents (regorafenib, bevacizumab, and fluorescent CDs) in one liposome. Compared with the existing targeted drug delivery systems for TNBC, our BCR-NLP platform offers several distinct advantages. While liposomal formulations have been widely studied for TNBC treatment,^51,52^ and CDs-based targeted systems have been reported for TNBC imaging,^53,54^ active targeting by bevacizumab leads to the selectivity of carrier but also provides an additional anti-angiogenic effect that acts synergistically with regorafenib. Meanwhile, the incorporation of fluorescent CDs allows non-invasive tracking of drug delivery without the need for external contrast agents. This is the first time that these three components have been combined together against TNBC.

## Author contributions

Abolghasem Abbasi Kajani: conception, design, and writing of the study. Armita Aryanmanesh: analysis, design and writing of the study. The manuscript was written through contributions of all authors. All authors have given approval to the final version of the manuscript.

## Conflicts of interest

The authors declare no conflict of interest.

## Abbreviations

BC: Breast cancer
BCR-NLPs: Bevacizumab-functionalized nanoliposomes loaded with carbon dots and regorafenib
CDs: Carbon dots
CR-NLPs: CDs- and regorafenib-loaded NLPs
DLS: Dynamic light scattering
DMSO: Dimethyl sulfoxide
DPPC: 1,2-Dipalmitoylphosphatidylcholine
EDC: 1-Ethyl-3-(3-dimethylaminopropyl)carbodiimide
EPR: Enhanced permeability and retention
FBS: Fetal bovine serum
FDA: Food and Drug Administration
FTIR: Fourier transform infrared
HER2: Human epidermal growth factor receptor 2
HPLC: High-performance liquid chromatography
HR-TEM: High resolution transmission electron microscope
LC: Loading capacity
LE: Loading efficiency
*m*PDA: *m*-Phenylenediamine
MTT: 3-(4,5-Dimethylthiazol-2-yl)-2,5-diphenyltetrazolium bromide
NHS: *N*-Hydroxy succinimide
NLPs: Nanoliposomes
NPs: Nanoparticles
PA: Palmitic acid
PBS: Phosphate buffered saline
QY: Quantum yield
R-NLPs: Regorafenib-loaded NLPs
TFH: Thin-film hydration
TKI: Tyrosine kinase inhibitor
TNBC: Triple-negative BC
UV-vis: Ultraviolet-visible
VEGF: Vascular endothelial growth factor

## Supplementary Material

RA-016-D6RA03364D-s001

## Data Availability

Data will be made available on request. All data generated or analyzed during this study are included in this published article and its supplementary information (SI). Supplementary information: zeta potential and XRD pattern of CDs; zeta potential of NLPs, B-NLPs, and BCR-NLPs; and the microscopic imaging of MDA-MB-231 and HFB cells after exposure with different NLPs. See DOI: https://doi.org/10.1039/d6ra03364d.
