# Supplementary material for: Synthesis and characterization of bevacizumab-functionalized nanoliposomes loaded with regorafenib and fluorescent carbon dots for triple-negative breast cancer theranostics
Source: RSC Adv. 2026 May 26;16(31):28516–25. doi: 10.1039/d6ra03364d (PMC13213576; doi:10.1039/d6ra03364d)
Supplement: RA-016-D6RA03364D-s001 [file RA-016-D6RA03364D-s001.pdf]

Supplementary Information (SI) for RSC Advances.
This journal is © The Royal Society of Chemistry 2026

Synthesis and characterization of bevacizumab-functionalized nanoliposomes loaded with regorafenib and fluorescent carbon dots for triple-negative breast cancer theranostics

Armita Aryanmanesh, Abolghasem Abbasi Kajani*

Department of Biotechnology, Faculty of Biological Science and Technology, University of Isfahan, Isfahan, 81746-73441, Iran

* Corresponding author:

Abolghasem Abbasi Kajani

Department of Biotechnology, Faculty of Biological Science and Technology, University of Isfahan, Isfahan 81746-73441, Iran, Tel: +98-3137934401, Fax: +98-3137932342, Email: agh.abbasi@bio.ui.ac.ir , agh.abasi@gmail.com

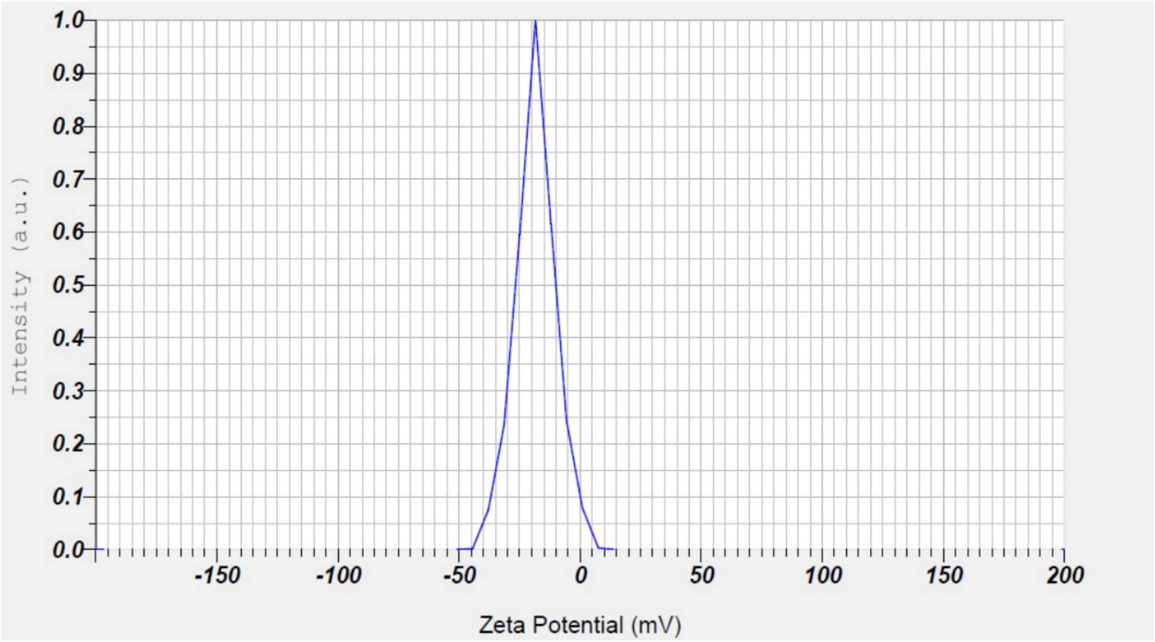

Fig. S1. Zeta potential of hydrothermally synthesized CDs using sucrose and mPDA.

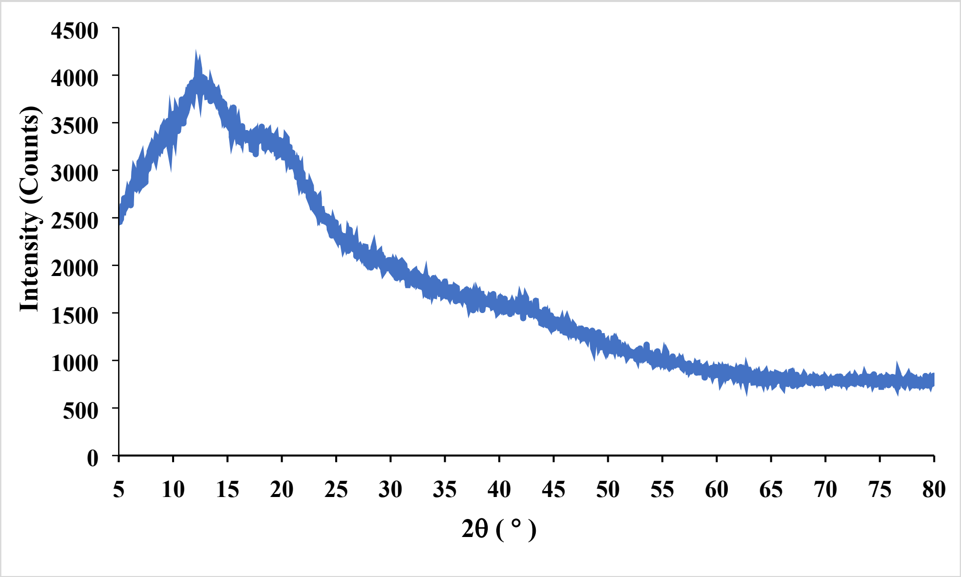

Fig. S2. The XRD pattern of CDs synthesized hydrothermally using sucrose and mPDA.

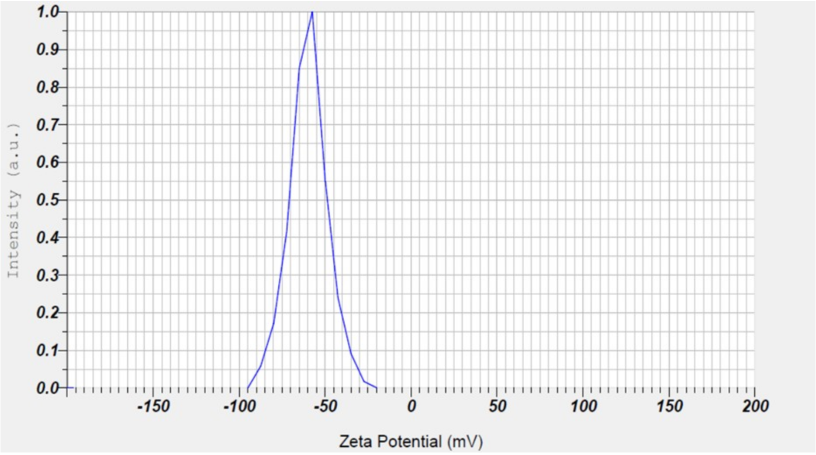

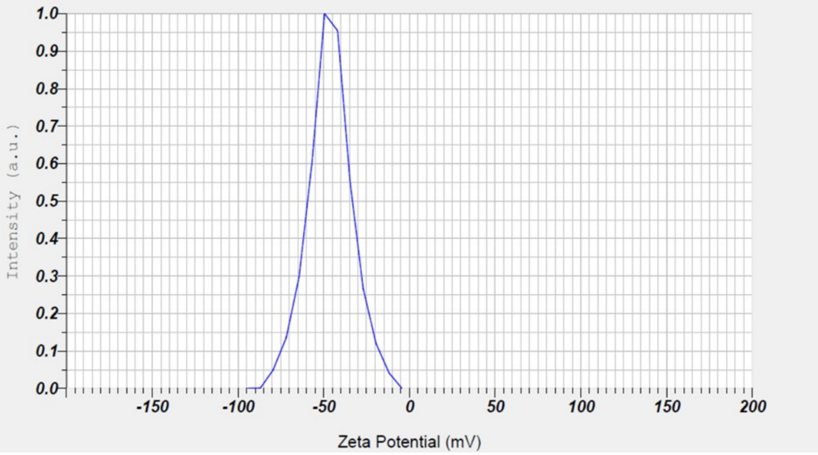

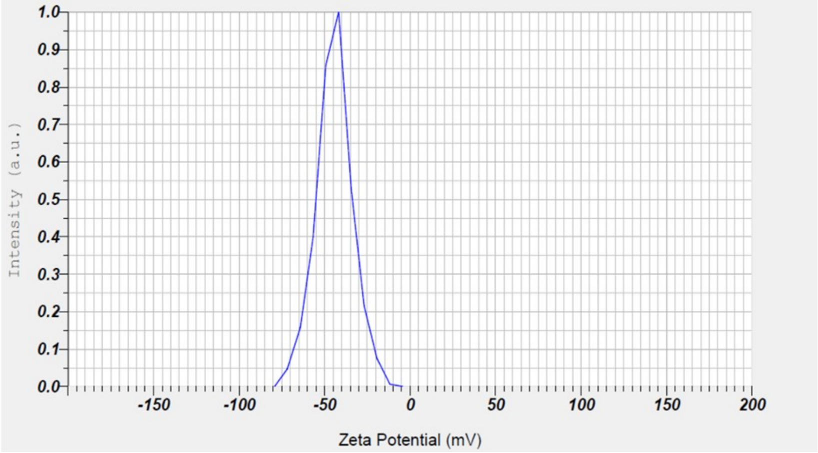

Fig. S3. Zeta potential of NLPs, B-NLPs, and BCR-NLPs.

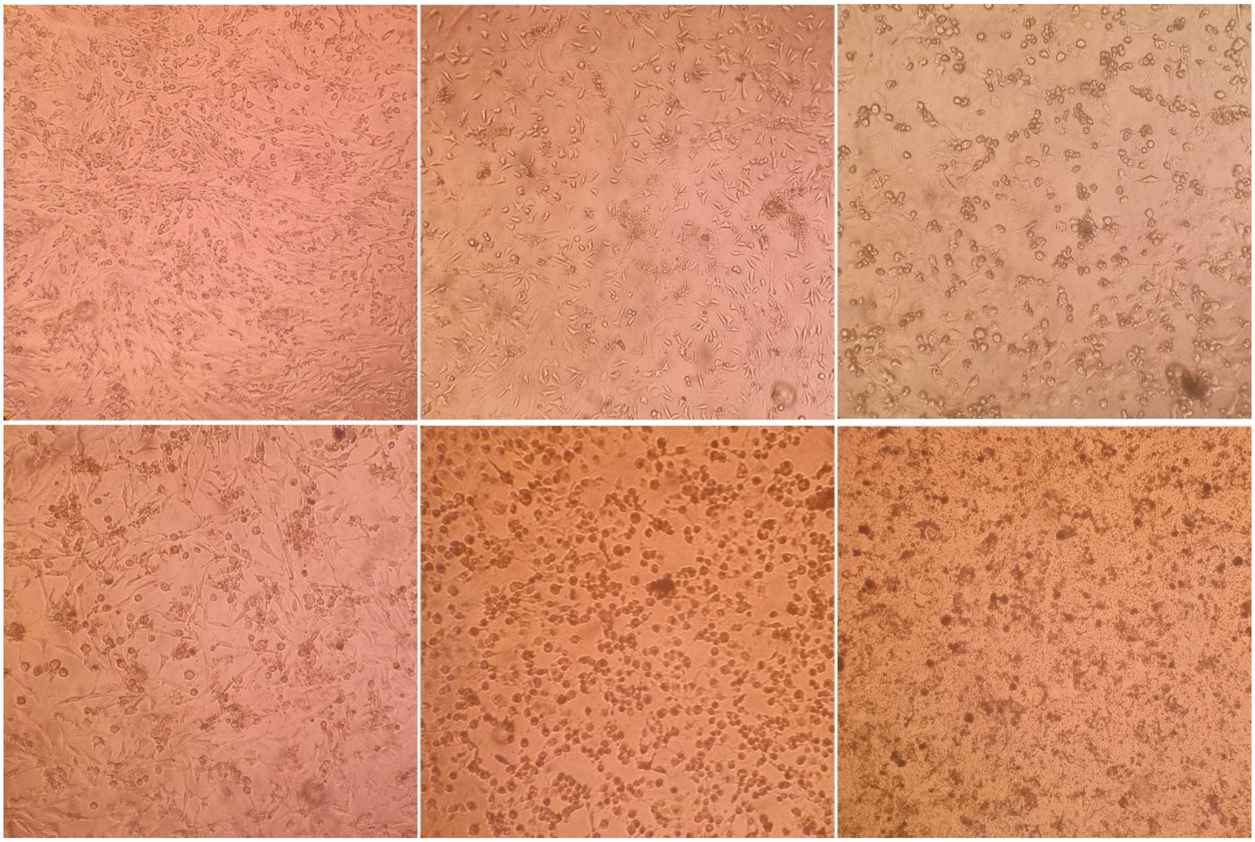

Fig. S4. The microscopic imaging of MDA-MB-231 cells after exposure with different NLPs. Left to right: Control, CDs, NLPs (up), and Regorafenib, CR-NLPs, and BCR-NLPs (down).

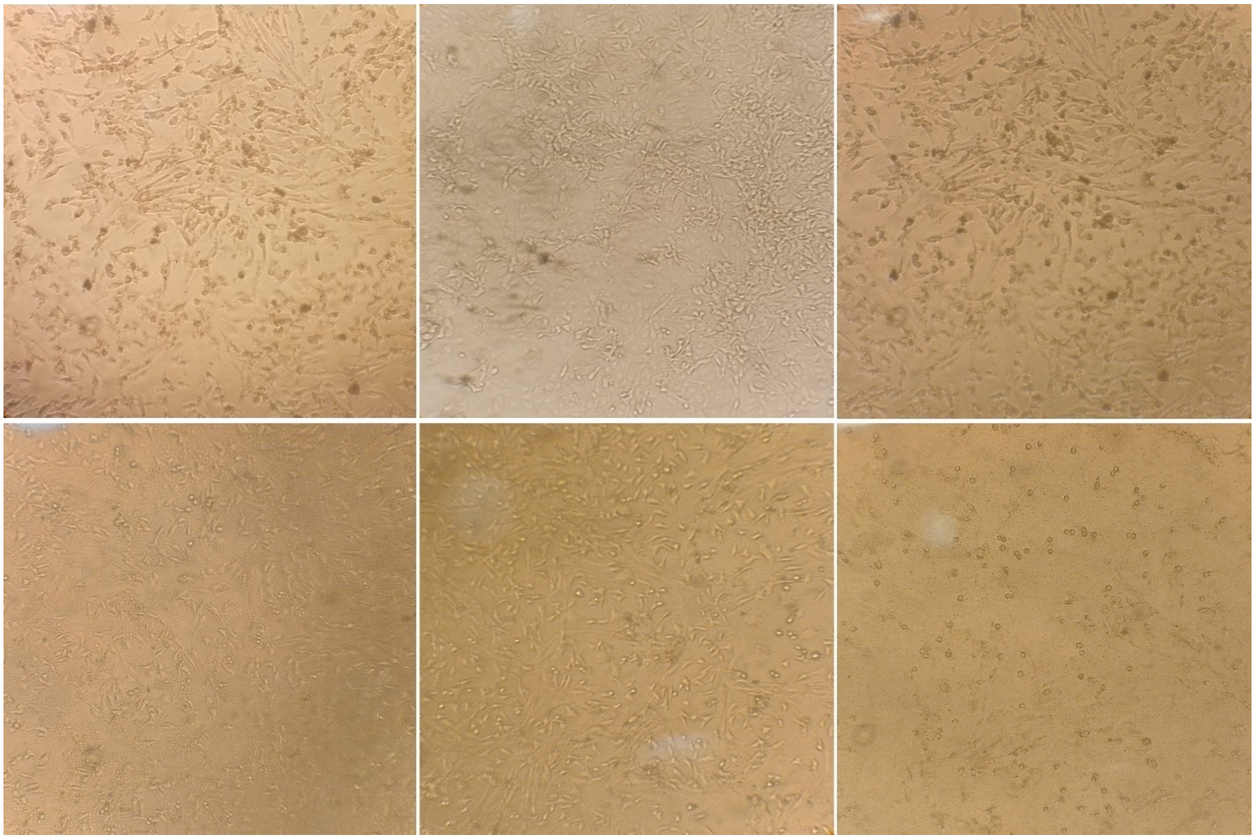

Fig. S5. The microscopic imaging of HFB cells after exposure with different NLPs. Left to right: Control, CDs, NLPs (up), and Regorafenib, CR-NLPs, and BCR-NLPs (down).
